# Transcription Analysis of the Chemerin Impact on Gene Expression Profile in the Luteal Cells of Gilts

**DOI:** 10.3390/genes11060651

**Published:** 2020-06-12

**Authors:** Karol G. Makowczenko, Jan P. Jastrzebski, Karol Szeszko, Nina Smolinska, Lukasz Paukszto, Kamil Dobrzyn, Marta Kiezun, Edyta Rytelewska, Barbara Kaminska, Tadeusz Kaminski

**Affiliations:** 1 Department of Animal Anatomy and Physiology, Faculty of Biology and Biotechnology, University of Warmia and Mazury in Olsztyn, Oczapowskiego 1A, 10-719 Olsztyn, Poland; karol.makowczenko@uwm.edu.pl (K.G.M.); karol.szeszko@uwm.edu.pl (K.S.); nina.smolinska@uwm.edu.pl (N.S.); kamil.dobrzyn@uwm.edu.pl (K.D.); marta.kiezun@uwm.edu.pl (M.K.); edyta.rytelewska@uwm.edu.pl (E.R.); barbara.kaminska@uwm.edu.pl (B.K.); 2 Bioinformatics Core Facility, Faculty of Biology and Biotechnology, University of Warmia and Mazury in Olsztyn, Oczapowskiego 1A, 10-719 Olsztyn, Poland; bioinformatyka@gmail.com (J.P.J.); lukasz.paukszto@uwm.edu.pl (L.P.); 3 Department of Plant Physiology, Genetics and Biotechnology, Faculty of Biology and Biotechnology, University of Warmia and Mazury in Olsztyn, Oczapowskiego 1A, 10-719 Olsztyn, Poland

**Keywords:** corpus luteum, gene expression, hormone, luteal phase, reproduction, transcription profile

## Abstract

Chemerin is a recently discovered adipokine that participates in the regulation of many physiological and disorder-related processes in mammals, including metabolism, inflammatory reactions, obesity, and reproduction. We investigated how chemerin affects the transcriptome profile of porcine luteal cells. The luteal cells were acquired from mature gilts. After the in vitro culturing with and without chemerin, the total RNAs were isolated and high-throughput sequencing was performed. Obtained datasets were processed using bioinformatic tools. The study revealed 509 differentially expressed genes under the chemerin influence. Their products take part in many processes, important for the functions of the corpus luteum, such as steroids and prostaglandins synthesis, NF-κB and JAK/STAT signal transducing pathways, and apoptosis. The expression of the *CASP3*, *HSD3B7*, *IL1B*, and *PTGS2* genes, due to their important role in the physiology of the corpus luteum, was validated using the quantitative real-time polymerase chain reaction (qPCR) method. The qPCR confirmed the changes of gene expression. Chemerin in physiological concentrations significantly affects the expression of many genes in luteal cells of pigs, which is likely to result in modification of physiological processes related to reproduction.

## 1. Introduction

Chemerin (CHEM) is an adipokine discovered more than 20 years ago, as a product of *TIG2*, later named as *RARRES2*. This substance was first identified during studies on the pathogenesis of psoriasis in 1997 as a product undergoing increased production under the influence of tazarotene [1]. To date, the occurrence of *RARRES2* mRNA was found, e.g., in the ovaries of women [2], mice [3], and rats [4]. In accordance to the Du and Leung studies, the swine CHEM amino acid sequence has the highest level of identity with respect to the human sequence (84%) among all of tested model animals – cattle, rats, and mice [5].

To date, three CHEM membrane receptors have been identified [6]—CMKLR1 (also named as ChemR23 or GPCR-DEZ), GPR1 and CCRL2. The first two mentioned receptors—CMKLR1 and GPR1—show a high structural similarity and they are ‘classic’ chemokine receptors, associated with G-proteins, which initiate intracellular signal transduction. The expression of CMKLR1 was reported in human ovarian cells [2] and the production of GPR1 mRNA was detected in the murine corpus luteum (CL) [7]. The effect of action of the third membrane receptor of CHEM, CCRL2, is regulation of ligand molecules availability and creation of chemokine or non-chemokine ligands gradient in in vivo [8] and in vitro [9] conditions, determining the direction of inflammatory reactions. It has been found that CCRL2 gene expresses in the human ovaries [2].

Recently, attention of researchers was also paid to contribution of CHEM to the influence on human and animal reproduction [2,7,10,11,12,13]. Yao and his team observed an increased accumulation of reactive oxygen species and an increase in granulosa cells (GC) apoptosis in the ovaries of mice in which upregulation of the expression of CHEM and CMKLR1-encoding genes was induced by a high-fat diet [14]. In human, mouse, and bovine GC, the influence of CHEM on the inhibition of steroidogenesis was found [2,7,12]. In addition, CHEM in rats triggers follicular growth arrest [15]. To date, the influence of CHEM on luteal cells (LC) has not been studied. Moreover, most studies analyzing the impact of CHEM on various physiological processes are carried out in humans or rodents, and often do not consider farm animals, including pigs—model organism of major importance to the human farming economy.

Our recent studies have shown the presence of all three CHEM-associated receptors in selected structures of the pig hypothalamus [16] and ovary [17] during the estrous cycle and early pregnancy, which allowed us to conclude that CHEM has a direct effect on the hypothalamic–pituitary–gonadal regulatory axis (HPG) by modifying the functioning of its upstream structures. We hypothesized that CHEM may have endocrine effects on downstream structures of the HPG axis, in particular on CL producing steroid hormones, significantly modifying reproductive processes in mammals. The aim of this study was to investigate the influence of CHEM on the transcriptomic profile of swine in vitro cultured luteal cells collected during the mid-luteal phase of the estrous cycle.

## 2. Materials and Methods

### 2.1. Experimental Animals and Tissue Collection

Five mature gilts (Large White × Polish Landrace; 7–8 months of age; body weight of 120–130 kg) obtained from a private breeding farm were used in this study. The gilts were on days 10–12 of the estrous cycle. Females were observed daily for behavioral estrus in the presence of boar. The day of onset of the second estrus was designated as day 0 of the estrous cycle. The phase of the estrous cycle was additionally confirmed based on ovarian morphology [18]. Ovaries collected immediately after slaughter of gilts were placed in ice-cold PBS supplemented with 100 IU/mL penicillin and 100 µg/mL streptomycin and transported to the laboratory on ice within 1 h for in vitro cell cultures preparation. The experiments were carried out in accordance with the ethical standards of the Animal Ethics Committee at the University of Warmia and Mazury in Olsztyn.

### 2.2. Isolation of the Luteal Cells and in Vitro Cell Cultures

Luteal cells were isolated using the method described by Kaminski et al. [19]. Dissected from ovaries corpora lutea on days 10–12 of the cycle (the mid-luteal period of fully functional CL) were minced into small fragments and dispersed in F-12 medium containing BSA (1%) and antibiotics. Corpora lutea were enzymatically dissociated in 0.125% trypsin solution (4–6 times) at 38 °C, centrifuged (300× *g*, 10 min, 21 °C), and washed three times. Isolated LC were filtered through nylon mesh (75 µm) and resuspended in fresh F-12 medium. The cells were counted using a hemocytometer, and their viability (≈90%) was determined by 0.4% trypan blue dye exclusion.

Luteal cells (2 × 10^6^/2 mL medium) were resuspended in F-12 medium enriched with FCS (20%), BSA (1%), and antibiotics, and pre-incubated for 48 h in a humidified incubator with 95% air and 5% CO_2_ atmosphere. The serum-containing medium was discarded, and the cells were washed using serum-free F-12 medium. After washing, LC were cultured for 24 h in F-12 medium with BSA (1%) and antibiotics, with or without recombinant human CHEM in concentration 200 ng/mL of medium. The concentration of the factor was selected on the basis of its relatively high level in women and porcine blood plasma [16,20].

### 2.3. RNA Isolation and High-Throughput Sequencing

Total RNA was extracted from in vitro cell cultures using RNeasy Mini Kit (Qiagen, Germantown, MD, USA) with DNase (RNase-free DNase Set, Qiagen, Germantown, MD, USA), according to manufacturer’s recommendations. The RNA quantity (wavelength 260 nm, A_260_) and purity (A_260_/A_280_) was assessed spectrophotometrically (Infinite M200 Pro, Tecan, Männedorf, Switzerland). Integrity of the total RNA was validated by electrophoresis on 1% agarose. Isolated RNA was stored at −80 °C for sequencing library preparation.

The sequencing library of template molecules suitable for following cluster generation was prepared using total RNA (from each sample) according to Shen et al. [21]. Firstly, the RNA solutions were purified from rRNA with use of Ribo-Zero rRNA Removal Kit (Illumina, San Diego, CA, USA). rRNA-depleted RNA was used to prepare strand-specific libraries using the TruSeq Stranded mRNA Library Prep Kit (Illumina, San Diego, CA, USA), following manufacturer’s protocol. Briefly, RNA was fragmented, ds-cDNA was synthesized replacing dTTPs with dUTPs in the reaction solution used in second strand cDNA synthesis. The obtained ds-cDNA fragments went through an end repair and A-tailing processes. Finally, specific adaptors were ligated to the obtained cDNA fragments. Polymerase chain reaction amplification was performed to enrich cDNA libraries.

The transcriptome high-throughput sequencing (RNA-Seq) of obtained cDNA libraries was performed on the NovaSeq 6000 platform (Illumina, San Diego, CA, USA) to generate 2 × 150 bp paired-end reads, with assumed minimal sequencing depth—100 million reads per sample.

### 2.4. In Silico Analyses

#### 2.4.1. Row Reads Pre-Processing and Differentially Expressed Genes Processing

**Figure 1:**
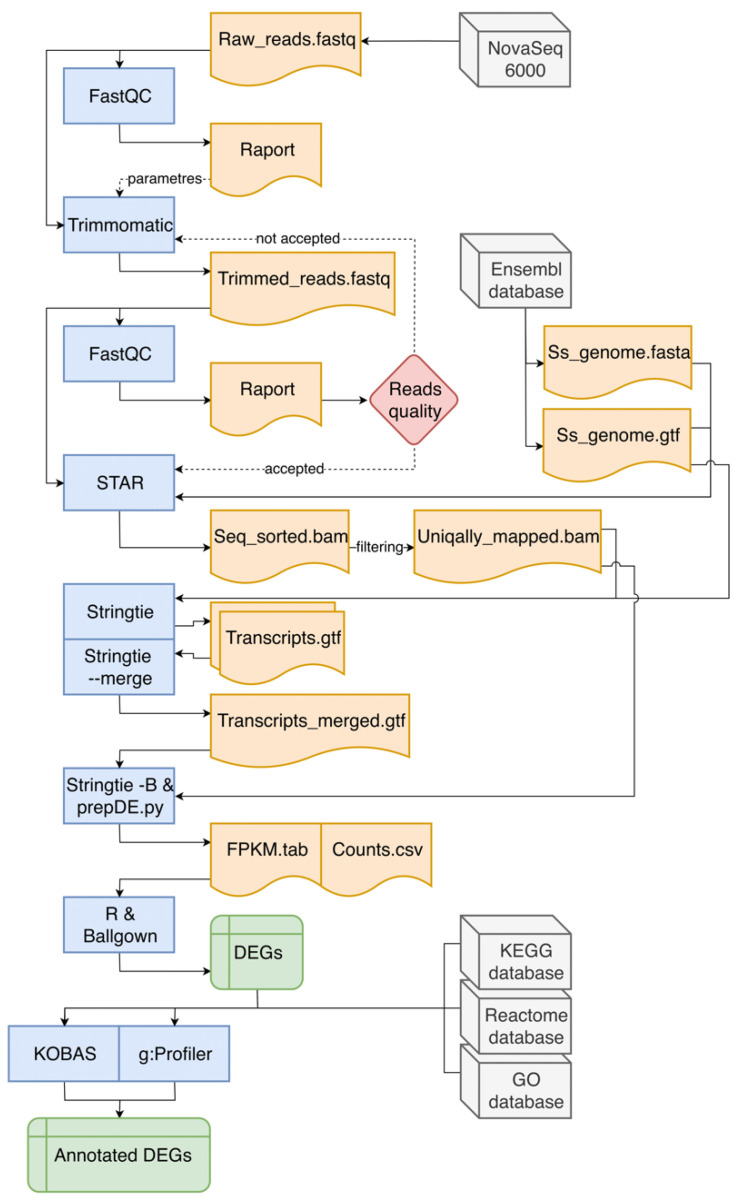
The course of in silico analyses. In the flowchart, blue blocks indicate used bioinformatic software, grey blocks represent databases (including the source of raw reads). Orange blocks symbolize input and output files necessary to run subsequent programs or tools. Green rectangles represent the result files that are the main effect of the entire data pipeline, which contain differentially expressed genes (DEGs). The red diamond symbolizes the decision-making process. Continuous arrows indicate the main direction of data flow, while intermittent arrows indicate the indirect influence on the analysis process.

This stage of analysis was performed accordingly to Pertea et al. [22] with significant modifications (Figure 1). Row reads obtained from sequencing were subjected to quality control, using FastQC v0.11.8 software [23]. Adapters and low-quality regions of row reads were trimmed with use of Trimmomatic v0.38 program [24]. After re-checking the quality and adapter content of processed reads, they were mapped with the use of STAR mapper v2.6.1d [25] to the *Sus scrofa* v11.1.91 reference genome downloaded from Ensembl database [26]. The number of reads mapped to exonic, intronic, untranslated regions (UTRs), or intergenic regions was quantified using the CollectRnaSeqMetrics tool in the Picard v2.21.1 software [27]. Principal component analysis (PCA) and Euclidean distances between samples analysis were performed using *ggplot2* library [28] and self-developed R script [29], to assess the overall similarity between transcriptomic profiles of RNA samples derived from CHEM-treated and non-CHEM-treated cells. StringTie aligner v1.3.5 [30] was used to enrich annotation of transcripts and prepare the Ballgown input files, with the fr-firststrand (*--rf*) enabled parameter. Counts per gene were computed on the basis of BAM files (alignments obtained from STAR) and GTF files (annotations from StringTie) using the *prepDE* python script with the Ballgown software [31]. Row reads were deposited in the European Nucleotide Archive database under the common project accession number—PRJEB35892.

Statistical analyses of differentially expressed genes (DEGs) for protein-coding RNA under the influence of CHEM was performed using Ballgown [31], dedicated Bioconductor v3.8 [32] package prepared for the R environment v3.5.2 [33], with the following operating parameters: *q*-value < 0.05 and |log_2_FC| ≥ 0.5.

#### 2.4.2. Functional Annotation of Differentially Expressed Genes

Functional analyses (gene ontology and pathway enrichment) were performed with use of g:Profiler [34] and KO-Based Annotation System v3.0 (KOBAS) [35] web tools related to Kyoto Encyclopedia of Genes and Genomes (KEGG) [36], Gene Ontology (GO) [37,38], and The Reactome Knowledgebase [39] databases.

### 2.5. Quantitative Real-Time PCR Validations

Quantitative real-time PCR (qPCR) was proceeded by cDNA synthesis using the same RNA as in the RNA-Seq (all CHEM-treated and control samples). A total of 500 ng of each total RNA sample was transcribed using the Omniscript RT Kit (Qiagen, Germantown, MD, USA), a mix of dNTPs and 0.5 μg oligo(dT) (Roche, Penzberg, Germany) in a total volume of 10 µL. The reaction was conducted at 37 °C for 1 h and was terminated by incubation at 93 °C for 5 min. qPCR was performed in technical duplicate for each sample with use of the 7300 PCR System (Applied Biosystems, Foster, CA, USA). The protocol assumed to use constitutively expressed *ACTB* and *GAPDH* as reference genes. Primer sequences for reference and target genes (*CASP3*, *HSD3B7*, *IL1B*, and *PTGS2*) were developed using Primer Express Software 3 (Applied Biosystems, USA). Quantitative real-time PCR mixtures with a final volume of 20 μL consisted of cDNA (40 μg), 400 nM of the primers, 12.5 μL of the Power SYBR Green PCR Master Mix (Applied Biosystems, USA), and RNase-free water. The primer sequences of all tested genes are listed in Table 1. Quantitative real-time PCR were performed under the following conditions: preliminary cDNA denaturation and enzymes activation at 95 °C for 10 min, followed by 40 cycles of denaturation at 95 °C for 15 s, annealing at 60 °C for 1 min, and elongation at 72 °C for 1 min. For *ACTB* and *HSD3B7* primers the annealing temperature was raised to 61 °C. Negative controls were prepared by replacing cDNA with water. All the samples were in duplicate. Calculation of the relative expression levels of validated genes was performed with use of the comparative cycle threshold method (ΔΔCT) and normalized using the geometrical means of the reference gene expression levels [40]. The results of qPCR were statistically processed in the R environment [33] with the use of one-factor ANOVA and were presented as mean values ± SEM. The results were regarded as statistically significant at *p*-value < 0.05.

## 3. Results

### 3.1. Overall Statistics of RNA-Seq Data and Mapping Results

The in vitro cultured LC sampled from five CHEM-treated groups and five control groups were used to create total cDNA libraries to explore the effect of CHEM treatment on the porcine LC transcriptome. The transcriptome high-throughput sequencing generated 1,154,494,264 raw paired-end reads. After pre-processing (minimum 90 bp length, Phred quality score > 30) and the removal of adapter sequences, 1,024,385,352 clean reads were obtained, of them 1,018,891,762 were mapped to the reference porcine genome (*S. scrofa* v11.1.91). On average, 96.52% of trimmed reads were uniquely mapped, 3.48% were mapped to multiple loci (Table 2).

**Figure 2:**
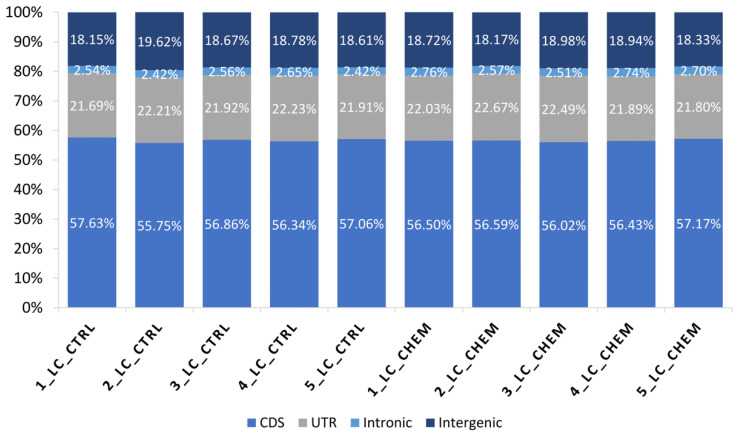
Percentage (%) of bases mapped to specific components of the pig reference genome among all samples: coding regions (CDS), untranslated regions (UTRs), intronic and intergenic regions.

In all libraries, 56.63% of processed reads were mapped to coding DNA sequence (CDS) regions, 22.08% were aligned to UTRs, 2.59% to introns, and 18.69% to intergenic locations (Figure 2). A total of 16,612 transcriptionally active regions (TARs) were identified in LC in at least half of the samples.

**Figure 3:**
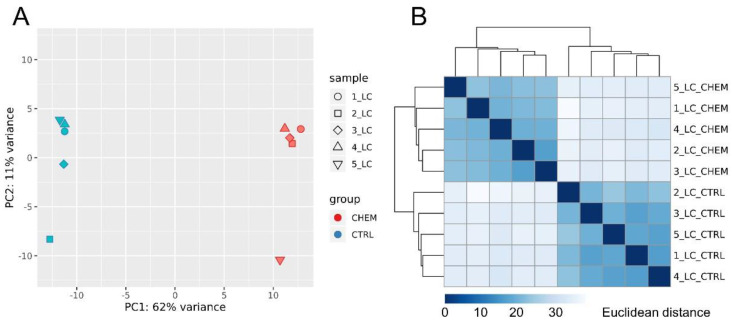
(**A**) Principal component analysis (PCA) of all differentially expressed genes. PCA plot was generated using the ggplot2 R library. (**B**) Sample-to-sample distance matrix. The color intensity means the distance, where dark blue is the lowest distance (the diagonal squares have distance 0) and the light blue (almost white) means high Euclidean distance. Heatmap showing the Euclidean distances between the samples was generated with use of the DESeq2 package.

Principal component analysis revealed a high degree of differentiation in gene expression profiles between research and control samples (Figure 3A). The calculated Euclidean distances showed high consistency of the expression profiles, both within the control group and the experimental group (Figure 3B).

### 3.2. Differentially Expressed Genes

**Figure 4:**
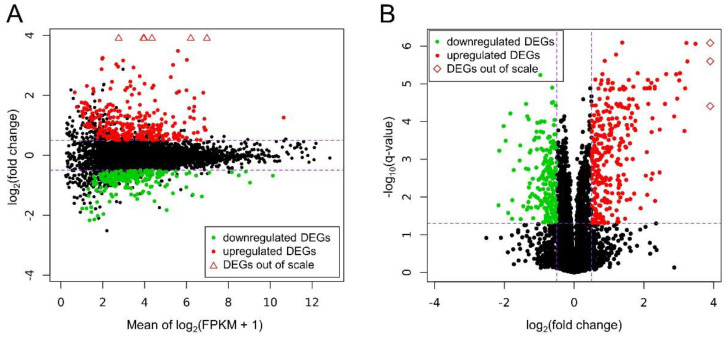
(**A**) MA plot shows the logarithmic values of fold changes (Y axis) and mean of logarithmic (fragments per kilobase per million reads mapped (FPKM) + 1) values (X axis) for comparing CHEM-treated and control libraries. (**B**) Volcano plot presents the logarithmic values of fold changes (X axis) plotted against negative logarithmic *q*-values (Y axis). On both plots each point is defaultly colored black, red dots represent differentially expressed genes (DEGs) upregulated under the CHEM treatment and green dots show downregulated DEGs with significant *q*-value < 0.05. Triangles symbolize DEGs above the visible range, and rhomboids symbolize DEGs to the right of the visible range. Purple dashed lines symbolize cut-off levels.

**Figure 5:**
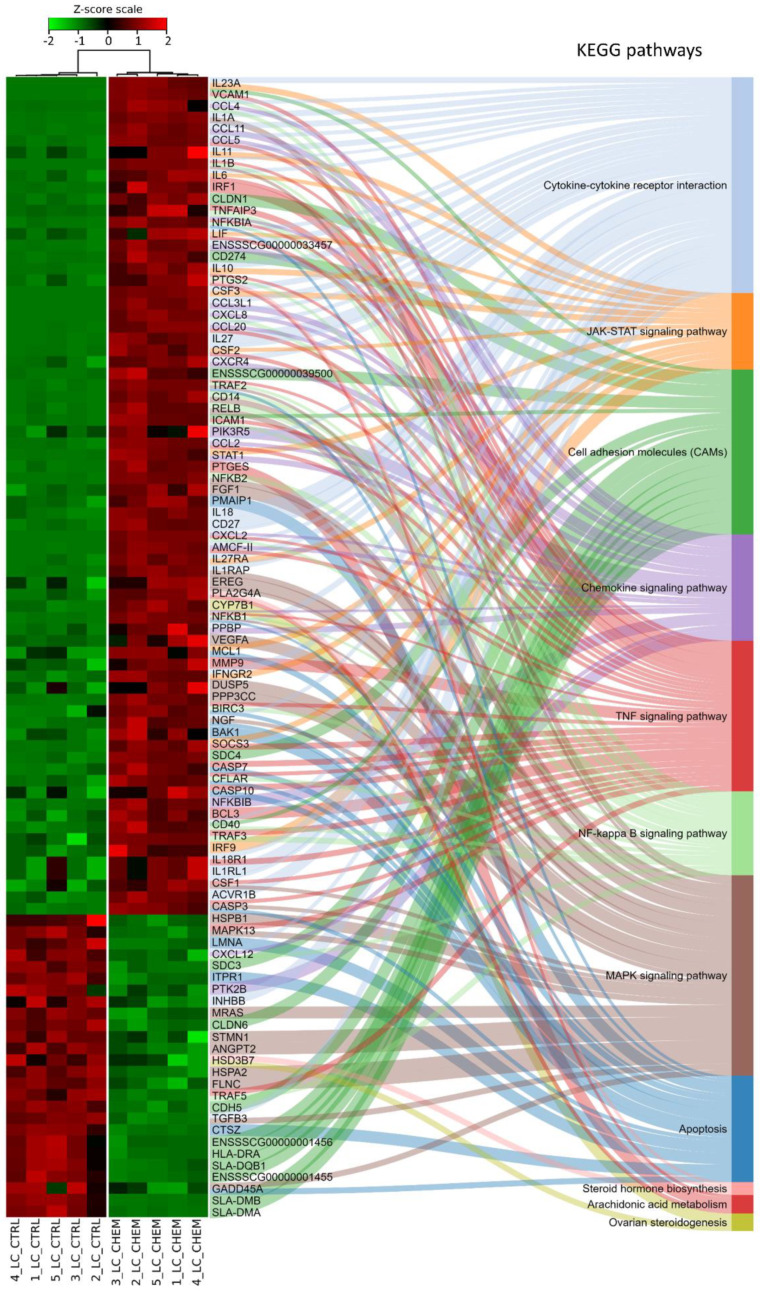
Heatmap of expression data for 98 differentially expressed genes and their participation in metabolic pathways from the Kyoto Encyclopedia of Genes and Genomes (KEGG) database. Genes were ordered according to the dendrogram (not shown) of similarity of their expression profiles calculated on the basis of the respective Z-scores. Samples marked with CTRL belong to the control group, while CHEM were treated with chemerin.

Ballgown-based analysis revealed that 509 genes (721 transcripts) showed statistically significant differences in expression between CHEM-treated and control samples (Table S1). The significant changes in gene expression profiles of in vitro cultured LC treated with CHEM are visualized in Figure 4. The 98 DEGs involved in metabolic pathways relevant for physiological functions of the corpus luteum (described below) are presented in a heatmap (Figure 5). Among all DEGs, 301 were upregulated and 208 were downregulated in the CHEM-treated group (Supplementary Table S1), and log_2_FC values ranged from 5.45 (*IL1B*) to −2.17 (*PANK1*). The 10 most upregulated genes were *IL1B*, *CSF3*, *CCL3L1*, *CXCL8*, *CCL20*, *CXCL2*, *ENSSSCG00000008954*, *AMCF-II*, *ACOD1*, and *IL23A*. The 10 most downregulated genes were *PANK1*, *SLA-DMA*, *ENSSSCG00000031640*, *SLA-DMB*, *ENSSSCG00000016725*, *PTGFR*, *FMNL2*, *ANKRD1*, *LYZ*, and *KCNK3* (Table S1).

### 3.3. Functional Genes Analysis

**Figure 6:**
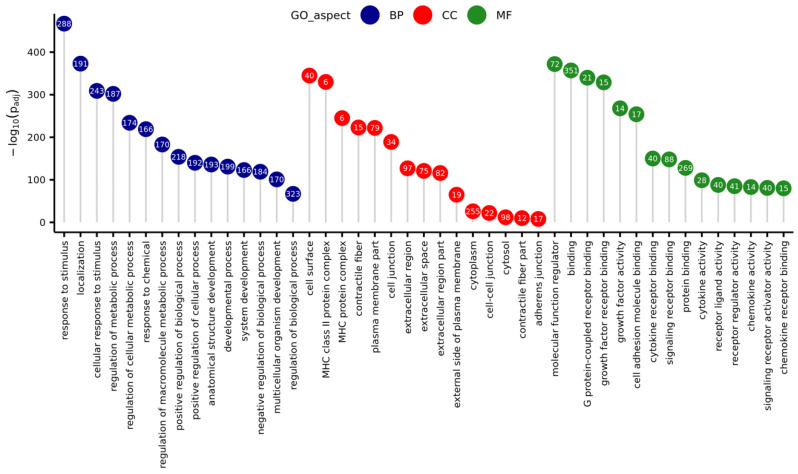
Lollipop chart with 15 best (with lowest *p*-value) Gene Ontology (GO) terms for every GO aspect (Biological Process—BP, Cellular Component—CC, Molecular Function—MF), sorted due to descending negative logarithmic adjusted *p*-value of enrichment analysis. Numbers in circles represent the number of differentially expressed genes matched to a specific term.

The GO enrichment analysis demonstrated that in the group of all DEGs, a total of 457 were associated with three aspects: biological process (BP), molecular function (MF), and cellular component (CC) GO terms (Figure 6). The BP aspect encompassed DEGs enriched to 407 statistically significant GO terms (*p*_adj_ < 0.05), for example: ‘cellular response to stimulus’, ‘regulation of signal transduction’, ‘immune system process’, ‘programmed cell death’, ‘response to cytokine’, ‘cytokine production’, and ‘I-kappaB kinase/NF-kappaB signaling’. Within the MF aspect, DEGs were enriched to 18 functions, for example ‘cytokine receptor binding’, ‘receptor regulator activity’, ‘growth factor receptor binding’, and ‘CCR chemokine receptor binding’. In the CC aspect, there were 15 significant GO terms, for example ‘extracellular region’, ‘cell surface’, ‘cytoplasm’, and ‘cell-cell junction’.

A subsequent KEGG enrichment classification revealed that 286 DEGs significantly affected by CHEM, had a statistically significant (*p*_adj_ < 0.05) impact on 51 biological pathways. Among all selected metabolic pathways, 32 were directly related to pathological processes such as carcinogenesis, autoimmune diseases, bacterial, viral infections, and those initiated by prions and protists. Due to the lack of relation with the studied tissue and the aim of understanding the physiological influence of CHEM on LC, these pathways were not analyzed. We investigated the impact of DEGs on the following signal pathways: ‘TNF signaling pathway’, ‘Cytokine-cytokine receptor interaction’, ‘NF-kappa B signaling pathway’, ‘Chemokine signaling pathway’, ‘MAPK signaling pathway’, ‘Cell adhesion molecules (CAMs)’, and ‘Apoptosis’ (Figures S1–S7). Additionally, following pathways, important for the CL functioning, that did not reach statistical significance during KEGG enrichment, were examined: ‘Steroid hormone biosynthesis’, ‘Ovarian steroidogenesis’, ‘Arachidonic acid metabolism’, and ‘JAK-STAT signaling pathway’ (Figures S8–S11).

The Reactome Knowledgebase enrichment analysis showed a significant contribution of 261 DEGs from the set obtained during this study in 19 molecular pathways, for example ‘Cytokine signaling in immune system’, ‘Programmed cell death’, ‘TAK1 activates NFkB by phosphorylation and activation of IKKs complex’, ‘Interleukin-1 signaling’, and ‘Chemokine receptors bind chemokines’.

### 3.4. Quantitative Real-Time PCR Validations

**Figure 7:**
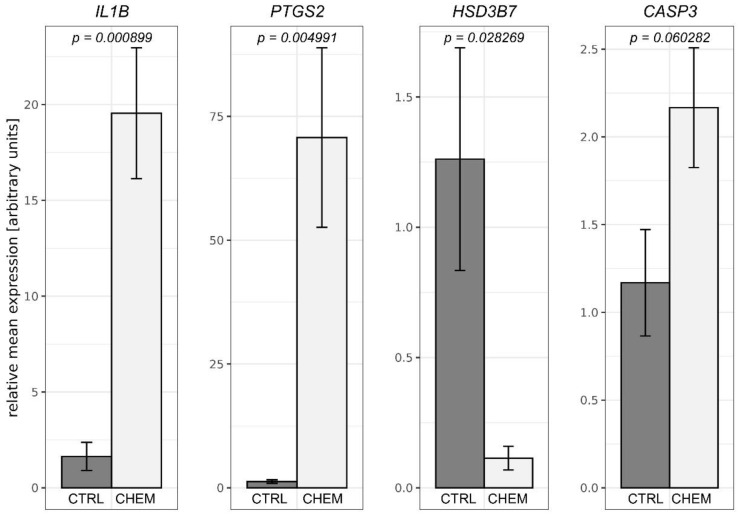
Quantitative real-time PCR validations of RNA-Seq results performed for selected differentially expressed genes—interleukin 1β (*IL1B*), prostaglandin-endoperoxide synthase 2 (*PTGS2*), hydroxy-δ-5-steroid dehydrogenase, 3 β- and steroid δ-isomerase 7 (*HSD3B7*) and caspase 3 (*CASP3*). Above the bars, precise *p*-values obtained from statistical analysis of the results are written. CTRL is a control group and CHEM is a chemerin-treated group.

To validate the obtained RNA-Seq results, four DEGs were selected for qPCR. The qPCR expression patterns of *HSD3B7*, *IL1B*, *PTGS2* were in agreement with RNA-Seq results. Changes in levels of *CASP3* expression between CHEM-treated and control samples did not reach the statistical significance threshold (*p*-value = 0.06), but nevertheless indicated the trend that had been previously revealed during the analysis of RNA-Seq data. Results of qPCR mostly confirmed the veracity of the high-throughput methods used in the present study (Figure 7).

## 4. Discussion

The transcriptome high-throughput sequencing was applied to identify the global transcriptome profile of treated and non-treated in vitro-cultured porcine luteal cells, acquired on days 10–12 of the estrous cycle. During this study, we observed the statistically significant impact of CHEM on changes of expression levels of 509 genes, whose products are involved in several processes important for functioning of porcine LC, and thus CL.

Previous studies evidenced a significant impact of CHEM on the ovarian cells of other species, such as humans [2,43], mice [7], rats [44], and cattle [12]. Nevertheless, the cited studies did not analyze the effects of CHEM on the LC at the transcriptional level, using deep sequencing techniques that allow certain relationships to be found with greater probability and sensitivity. The authors would like to point out that in accordance to the literature data, gene expression affects at least 40% [45,46] of the variability of protein produced in mammalian cells. However, as stated by Li and co-workers [47], taking into account experimental errors, changes at the mRNA level explain up to 84% of the variance at the protein level.

Nuclear factor κB, the subunits’ production of which is strongly modified by CHEM in the porcine LC, is a protein complex controlling transcription of proteins, which contribute to the inflammatory response, through triggering synthesis of proinflammatory factors, regulating inflammatory reaction course, leukocyte recruitment, and apoptosis or cell survival [48]. Signal transduction can be carried out by ‘canonical’ pathway, activated by TNFα and IL1 or by ‘alternative’ pathway, in which the signal is activated by, among others, a CD40 membrane receptor [48]. Despite the lack of statistically significant change in the expression of TNFα-coding genes under the influence of CHEM, the level of *TNFAIP3* mRNA produced by LC increased significantly (FC = 4.6). Furthermore, *TNIP1* was 2.1-fold upregulated. TNF-receptor associated factor proteins are involved in the initial signal transduction in the ‘alternative’ NF-κB pathway. The expression of *TRAF2* (FC = 2.6) was also modified by CHEM. Zmijewska and colleagues proved that IL1B in CL is secreted not only by macrophages but also by LC [49]. In CHEM-treated samples, we observed a 43.6-fold increase in the production of *IL1B* transcripts, an 8.1-fold increase in the production of *IL1A* transcripts, and a 2.0-fold increase in the production of *IL1RAP* transcripts, in comparison to control samples. Moreover, IRAK2 mediates the process of the NF-κB pathway activation by IL1 molecules [50], and its mRNA was produced in increased quantities under the influence of CHEM (FC = 1.7). Some of the proteins constructing the NF-κB complex, such as *NFKB1* (FC = 1.8), *NFKB2* (FC = 2.3), *RELB* (FC = 2.5), *NFKBIA* (FC = 4.2), *NFKBIB* (FC = 1.6), *NFKBIE* (FC = 2.0), and *NFKBIZ* (FC = 2.8) were upregulated. Proteins encoded by *NFKB1* and *NFKBIA* participates in the ‘canonical’ pathway, whereas *RELB* and *NFKB2* take a part in the ‘alternative’ NF-κB pathway. Interestingly, Xia and co-workers suggested that the ‘canonical’ pathway of the NF-κB can be inhibited by RELB protein, which is able to regulate the stability of protein encoded by the *NFKBIA* gene [51]. The effect of activating the ‘canonical’ pathway is an increase in the expression of many genes, such as mentioned *IL1A*, *IL1B* and *IL18, PTGS2*, *PTGES*, *BIRC3*, *MCL1*, and *CFLAR*, discussed in detail below [52]. As a result of previous studies conducted by Luo and colleagues [53], and Przygrodzka and colleagues [54], it was found that activation of the NF-κB pathway occurs in LC at a late luteal stage of the estrous cycle and is associated with the acquisition of luteolytic sensitivity by CL. This implies the participation of CHEM in the process of early acquisition of luteolysis capacity by LC. On the other hand, Vince and co-workers showed that TRAF2 must recruit proteins encoded by *CIAP2* (also named as *BIRC3* or *HIAP1*; FC = 1.7) to properly activate the NF-κB pathway and to create resistance to apoptosis induction [55]. Overexpression of genes encoding both proteins in LC transcriptomes treated with CHEM allows to assume also anti-apoptotic action of the described pathway.

The JAK/STAT pathway takes part in the transduction of signals associated with cell differentiation, proliferation, migration, or apoptosis [56]. It consists of only a few components, such as membrane receptors with a characteristic domain capable of binding JAK proteins, JAKs capable of phosphorylation STAT proteins, and a group of STATs, which activated and homodimerized may act as transcription factors [57]. The results revealed 2.3-fold upregulation of the *STAT1* gene. More than 20 years ago, it was proven that STAT1 is an essential factor for the constitutive expression of, inter alia, *CASP3* described below [58]. It was previously stated that homodimerization of STAT1 proteins is induced by IFNγ [59]. We did not identify any statistically significant increase in IFNγ-coding gene expression, but we did notice the upregulation of one of its receptors—*IFNGR2* (FC = 1.8). Moreover, the expression of *IL18*, also known as IFNγ inducing factor-coding gene, and its receptor-coding gene (*IL18R1*) were, respectively, 2.1-fold and 1.5-fold upregulated. Tsuji and his colleagues discovered the production of IL18 and IL18R proteins directly by mouse LC, linking their observations to the paracrine and autocrine activity of these cells [60]. This may be an argument for probably more effective IFNγ action in the studied cells, despite the lack of a significant increase in the content of mRNAs. Furthermore, it has been confirmed that IFNγ may control porcine CL functions, including progesterone (P_4_) production [61,62]. Gene expression profiles also revealed differences in the content of *SOCS3* (1.7-fold upregulation under CHEM influence) mRNA produced. SOCS3 is a known regulator of JAK/STAT pathway, induced by this pathway activation [56].

The corpus luteum is the primary endocrine gland that directly regulates the functioning of the uterus and modulates its transformation during the estrous cycle. Corpora lutea perform their function mainly by producing P_4_, a steroid hormone that prepares the endometrium for embryo implantation and is responsible for maintenance of gestation. The key steroidogenic enzyme involved in the production of P_4_ by LC is 3β-HSD. We observed a significant decrease in the content of *HSD3B* mRNA produced under the influence of CHEM (FC = 0.60), and we confirmed the expression of *HSD3B* by qPCR. Surprisingly, Rytelewska and co-workers [63] observed an induction of P_4_ secretion by CHEM-treated in vitro-cultured porcine LC obtained on days 10–12 of the estrous cycle. The difference between the observed amount of *HSD3B* mRNA in porcine LC and the level of P_4_ secretion may result from the negative feedback loop. The *HSD3B* feedback regulation by P_4_ was previously mentioned in rat ovaries by de Galarreta and co-workers [64], and Tanaka and colleagues [65]. Similarly, downregulation of *HSD3B* was noticed in testicular Leydig cells under the influence of androgens [66,67]. As a result of this mechanism, a large amount of P_4_ produced by LC may directly inhibit the expression of *HSD3B*. Furthermore, Rytelewska and colleagues [63] conducted analyses using in vitro LC cultures (identical to those used in this study) of the CHEM effect on basal and luteinizing hormone or follicle-stimulating hormone and/or insulin-induced secretion of other essential steroid hormones, such as androstenedione (A_4_), testosterone (T), estrone (E_1_), and estradiol (E_2_). The mentioned study revealed that CHEM inhibits basal and induced E_2_ secretion, and exerts both stimulatory and inhibitory effects on basal and induced secretion of A_4_, T, and E_1_ (depending on the phase of the estrous cycle). According to Rytelewska and co-workers [63], CHEM appears to be an important factor that modulates ovarian steroidogenesis in pigs, whereas its stimulatory or inhibitory effects on the secretion of steroid hormones may be due to the heterogeneity of factors regulating ovarian functions, possible interactions between these factors, and specific processes related to the ovarian physiology during different phases of the estrous cycle.

We found upregulation of *cPLA2* (also named *PLA2G4B*; 1.9-fold increase), *PTGS2* (also named *COX2*; 3.2-fold increase), and *PTGES* (2.3-fold increase). Enzymes encoded by these genes create the complete metabolic pathway, during which the arachidonic acid substrate is transformed into the final product, which is luteoprotective PGE_2_ [68]. In mammals, PGE_2_ is mainly responsible for the ovulation process and the subsequent luteinization of the ovarian follicle [69], which is also related to an increase in the production of *HSD3B* mRNA [70] and further in P_4_ secretion [71]. This assumption coincides with the observations of Zmijewska and co-workers [49] on the influence of IL1B on the expression of *PTGS2* and *PTGES* genes, and thus PGE_2_ secretion. It is also probable that such a high increase in observed *IL1B* gene expression is the result of positive feedback loop via the NF-κB signal transduction pathway and upregulation of *PTGS2* in swine LC, as was previously observed in human intervertebral disc cells [72]. Interestingly, it was affirmed that expression of the *PTGES* gene in swine CL does not directly correspond with intraluteal level of PGE_2_ due to its transport to ovary from conceptuses and the uterus, in order to rescue luteal function during the maternal recognition of pregnancy [73]. We observed 7.3-fold upregulation of *PKIB*. The protein encoded by this gene is an inhibitor of PKA enzymes family involved in the transduction of signals induced among others by PGE_2_. This observation may be a direct symptom of a strong influence of CHEM on PGE_2_ generation by porcine LC.

Programmed death of LC is one of the integral elements of CL lifespan and is directly related to the process of structural regression of CL [74]. During this research, we noticed increased expression of genes encoding apoptosis initiator caspase (*CASP10* (FC = 1.6)) and executioner caspases (*CASP3* (FC = 1.4) and *CASP7* (FC = 1.6)) in samples treated with CHEM, which usually indicates the beginning of apoptotic processes in the studied cells. Increased quantity of produced CASP3 protein or mRNA, a key executioner protease, could indicate an ongoing process of cellular apoptosis by both intrinsic and extrinsic pathways, which has been observed under the influence of CHEM in the ovarian cells of other mammals [7,14,15]. Validation of RNA-Seq results by qPCR confirmed a tendency to increase the quantity of *CASP3* mRNA produced in CHEM-treated samples in comparison to control samples. Due to the fact that obtained *p*-value was just above the assumed threshold of statistical significance, such a result cannot be considered as conclusive. Encoded by upregulated *BAK1* (FC = 1.7) and *PMAIP1* (also named Noxa protein; FC = 2.1) genes pro-apoptotic proteins, belonging to BCL2 family members, may form specific, for intrinsic apoptosis mitochondrial membrane, ‘permeability transition pores’ [75]. These pores may enable exit from mitochondria to cytosol molecules, that induce DNA defragmentation, formation of apoptosomes activating CASP9 (which in turn triggers CASP3), and formation of complexes suppressing the activity of apoptosis inhibitory proteins [74,76].

Nonetheless, cellular mechanisms to counteract programmed cell death are also significantly strengthened, as can be seen from the increase in the amount of produced *CFLAR* (also named *C-FLIP*; FC = 1.6), *MCL1*, *CIAP2* mRNAs. C-FLIP protein binding FADD and CASP8 and/or CASP10 proteins prevents formation and activation of the caspase cascade [77]. Long isoforms of MCL1 protein are able to bind many proteins from the BCL2 family, preventing the formation of mitochondrial ‘permeability transition pores’. The CIAP2 protein has the ability to regulate apoptosis by binding caspases, but also to modulate inflammatory signaling by ubiquitination of proteins involved in the NF-κB signal pathway [78]. Gene encoding PFKFB3 protein was 2.5-fold upregulated in CHEM-treated samples. This gene acts as a regulator of glucose metabolism within cells and has been associated with prevention of apoptosis [79]. Modifications of expression of the genes mentioned in this paragraph, induced by CHEM treatment on the mid-luteal LC, suggest that despite the occurrence of strong pro-apoptotic signals in cells there are also parallel mechanisms of opposite effect—pro-survival. We assume that despite the visible preparation of LC for programmed death, a multitude of anti-apoptotic processes inhibit the action of executive factors. It is possible that this phenomenon is directly related to the phase of the estrous cycle and the lack of fully developed luteolytic activity by the cells.

## 5. Conclusions

This study was the first experiment to demonstrate the impact of CHEM on the transcriptome profile of porcine LC during the mid-luteal phase of the estrous cycle. During this study we showed, for the first time, the impact of CHEM on NF-κB and JAK/STAT signal transduction pathways in mammalian LC. We observed changes in expression of apoptosis-associated genes which products may in a cell-specific manner affect the activation of both pro-survival and pro-apoptotic signaling pathways.

It is highly probable that CHEM at the physiological concentration may influence the sophisticated metabolic processes associated with gilt reproduction by regulation of P_4_ generation, increased production of pro-inflammatory factors, and preparation for luteal regression. As a result, deregulation of the ovarian–uterine interactions may result in prevention of embryo implantation, loss of pregnancy, or significant reduction in fertility. This research may be a prelude to further studies of the effect of CHEM on the swine reproductive system, as an important farm animal and as a model organism with a physiology like that of humans.

## Figures and Tables

**Table 1:** Primers used for the validation of RNA-Seq results.

Gene Symbol	Gene Description	Primers Sequences	Product Length	Reference
*IL1B*	Interleukin 1β	F: TTTGAAGAAGAGCCCATCATCC R: CCAGCCAGCACTAGAGATTTG	119 bp	[The present study]
*CASP3*	Caspase 3	F: GTGCTTCTAAGCCATGGTGAA R: CGGCAGGCCTGAATTATGAA	143 bp	[The present study]
*PTGS2*	Prostaglandin-endoperoxide synthase 2	F: ATGGGTGTGAAAGGGAGGAAA R: AAACTGATGGGTGAAGTGCTG	141 bp	[The present study]
*HSD3B7*	Hydroxy-δ-5-steroid dehydrogenase, 3 β- and steroid δ-isomerase 7	F: CTCGAAGCCAACGGAAGGA R: CCACGTTACCCACGTAGACC	193 bp	[The present study]
*ACTB*	β-actin	F: ACATCAAGGAGAAGCTCTGCTACG R: GAGGGGCGATGATCTTGATCTTCA	366 bp	[41]
*GAPDH*	Glyceraldehyde-3- phosphate dehydrogenase	F: CCTTCATTGACCTCCACTACATGG R: CCACAACATACGTAGCACCAGCATC	183 bp	[42]

Abbreviations: F—forward; R—reverse.

**Table 2:** Summary of the results of RNA sequencing, preprocessing and mapping of reads to the porcine reference genome. All numerical values are expressed in millions. CTRL are samples from control group, and CHEM are samples from chemerin-treated group.

Treatment			CTRL					CHEM		
Samples	1_LC	2_LC	3_LC	4_LC	5_LC	1_LC	2_LC	3_LC	4_LC	5_LC
Row reads	110.458	125.606	121.017	115.746	108.415	109.209	115.928	112.795	122.503	112.816
Processed reads	97.605	111.356	106.973	102.611	96.010	97.413	102.895	99.857	109.165	100.500
Mapped reads	97.057	110.805	106.363	102.009	95.54	96.93	102.244	99.362	108.613	99.97
Uniquely mapped	94.274	106.514	102.766	98.577	92.284	93.337	99.22	95.599	104.365	96.408
% of uniquely mapped	97.13%	96.13%	96.62%	96.64%	96.59%	96.29%	97.04%	96.21%	96.09%	96.44%
Multi-mapped	2.783	4.291	3.597	3.432	3.256	3.593	3.024	3.763	4.248	3.562

## Supplementary Materials

The following are available online at https://www.mdpi.com/2073-4425/11/6/651/s1, Table S1: Differentially expressed protein coding genes identified in porcine luteal cells after in vitro chemerin treatment, Figure S1: TNF signaling pathway with mapped expression data, Figure S2: Cytokine–cytokine receptor interaction with mapped expression data, Figure S3: NF-kappa B signaling pathway with mapped expression data, Figure S4: Chemokine signaling pathway with mapped expression data, Figure S5: MAPK signaling pathway with mapped expression data, Figure S6: Cell adhesion molecules with mapped expression data, Figure S7: Apoptosis pathways with mapped expression data, Figure S8: Steroid hormone biosynthesis pathways with mapped expression data, Figure S9: Ovarian steroidogenesis pathways with mapped expression data, Figure S10: Arachidonic acid metabolism pathway with mapped expression data, Figure S11: JAK/STAT signaling pathway with mapped expression data
