# Supplementary figures and images for: Transcription Analysis of the Chemerin Impact on Gene Expression Profile in the Luteal Cells of Gilts
Source: Genes (Basel). 2020 Jun 12;11(6):651. doi: 10.3390/genes11060651 (PMC7349926; doi:10.3390/genes11060651)

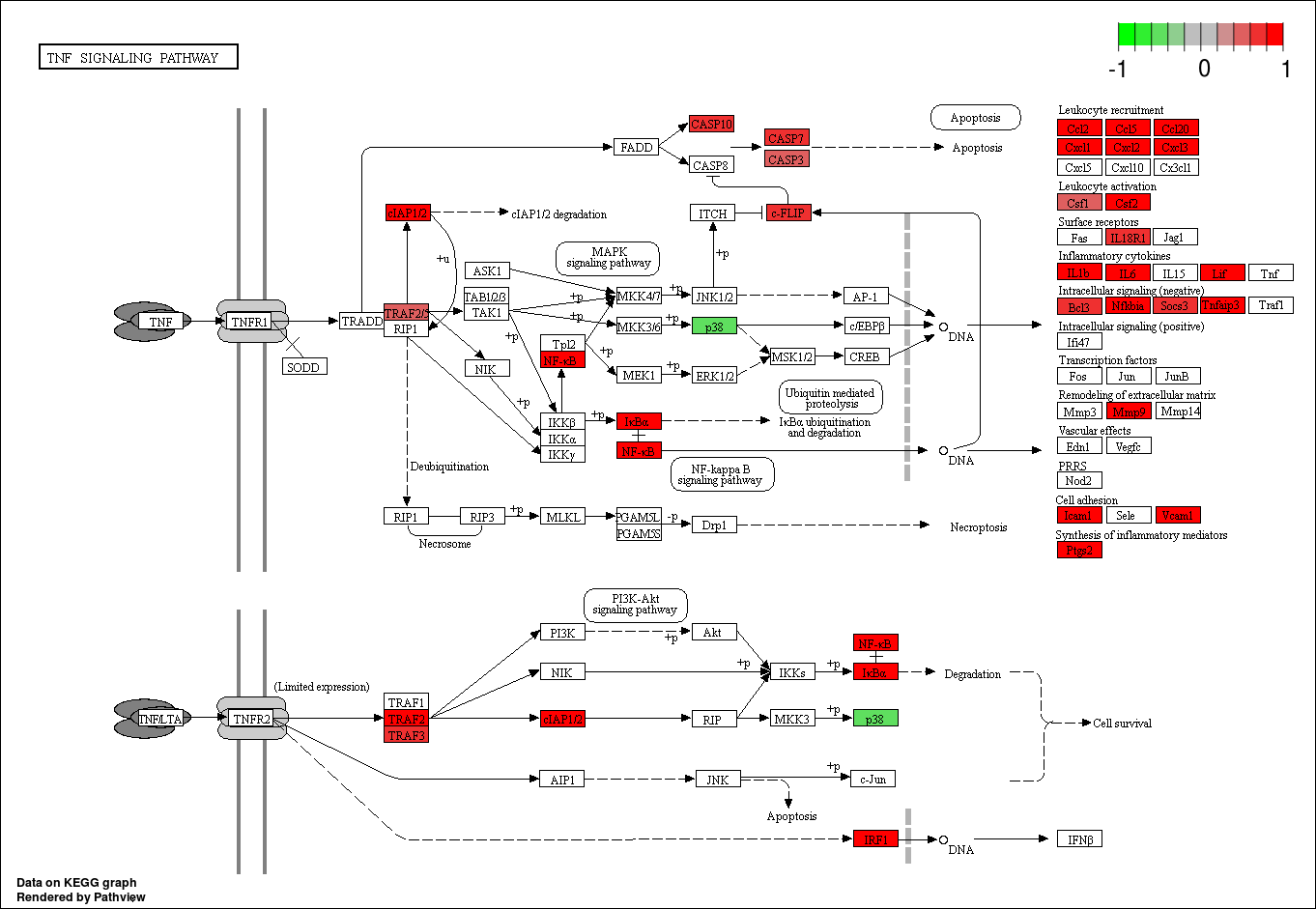

Supplement: Supplementary file 1 [file genes-11-00651-s001.zip › supplementary_materials/Figure_S1.png]

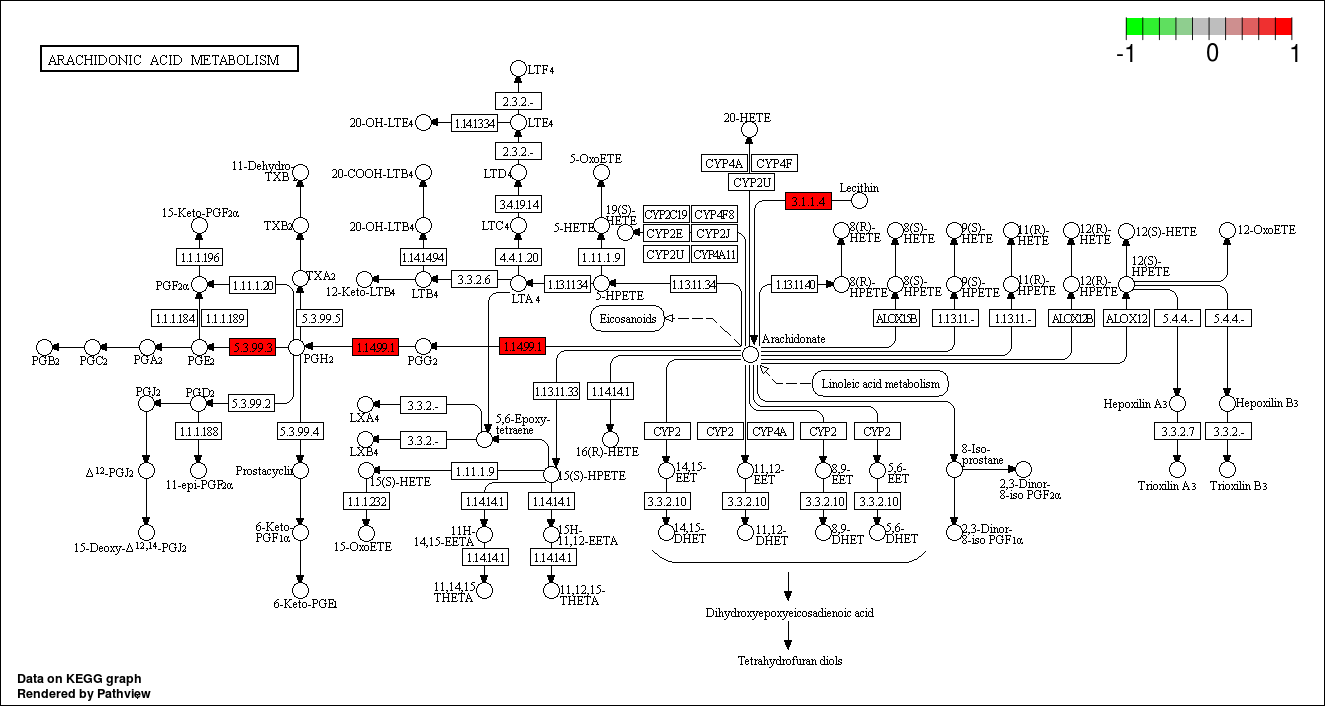

Supplement: Supplementary file 1 [file genes-11-00651-s001.zip › supplementary_materials/Figure_S10.png]

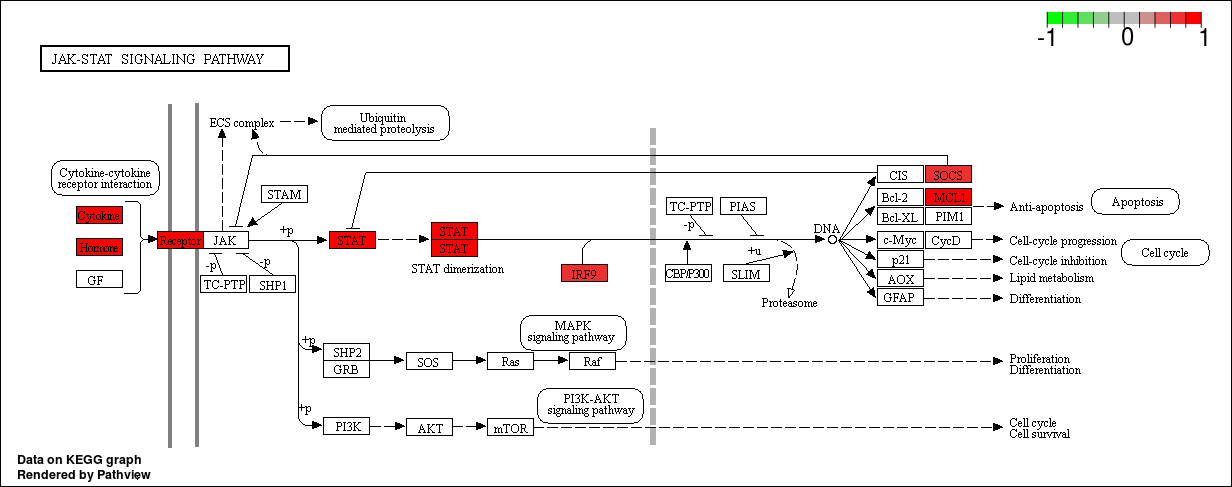

Supplement: Supplementary file 1 [file genes-11-00651-s001.zip › supplementary_materials/Figure_S11.png]

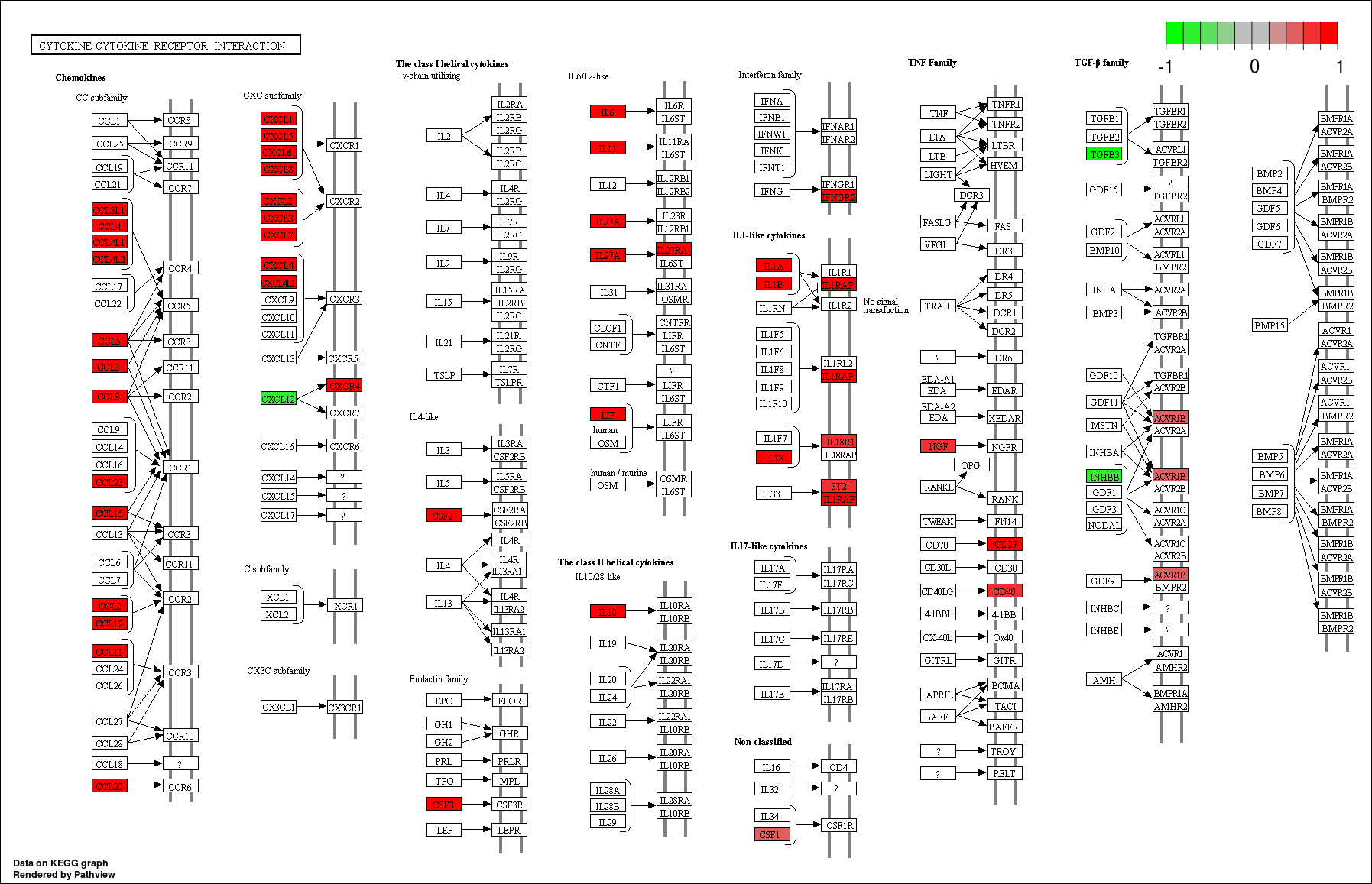

Supplement: Supplementary file 1 [file genes-11-00651-s001.zip › supplementary_materials/Figure_S2.png]

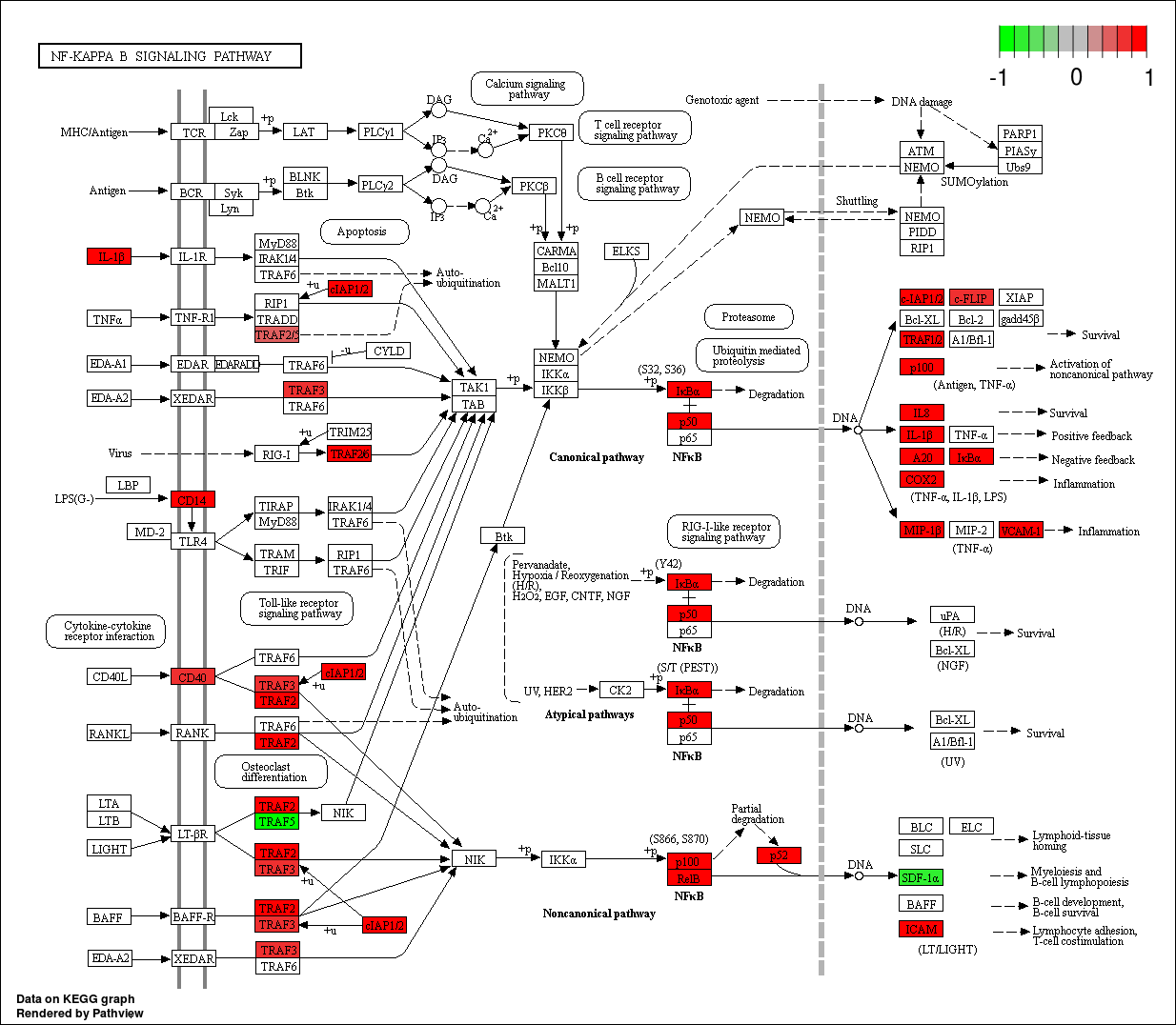

Supplement: Supplementary file 1 [file genes-11-00651-s001.zip › supplementary_materials/Figure_S3.png]

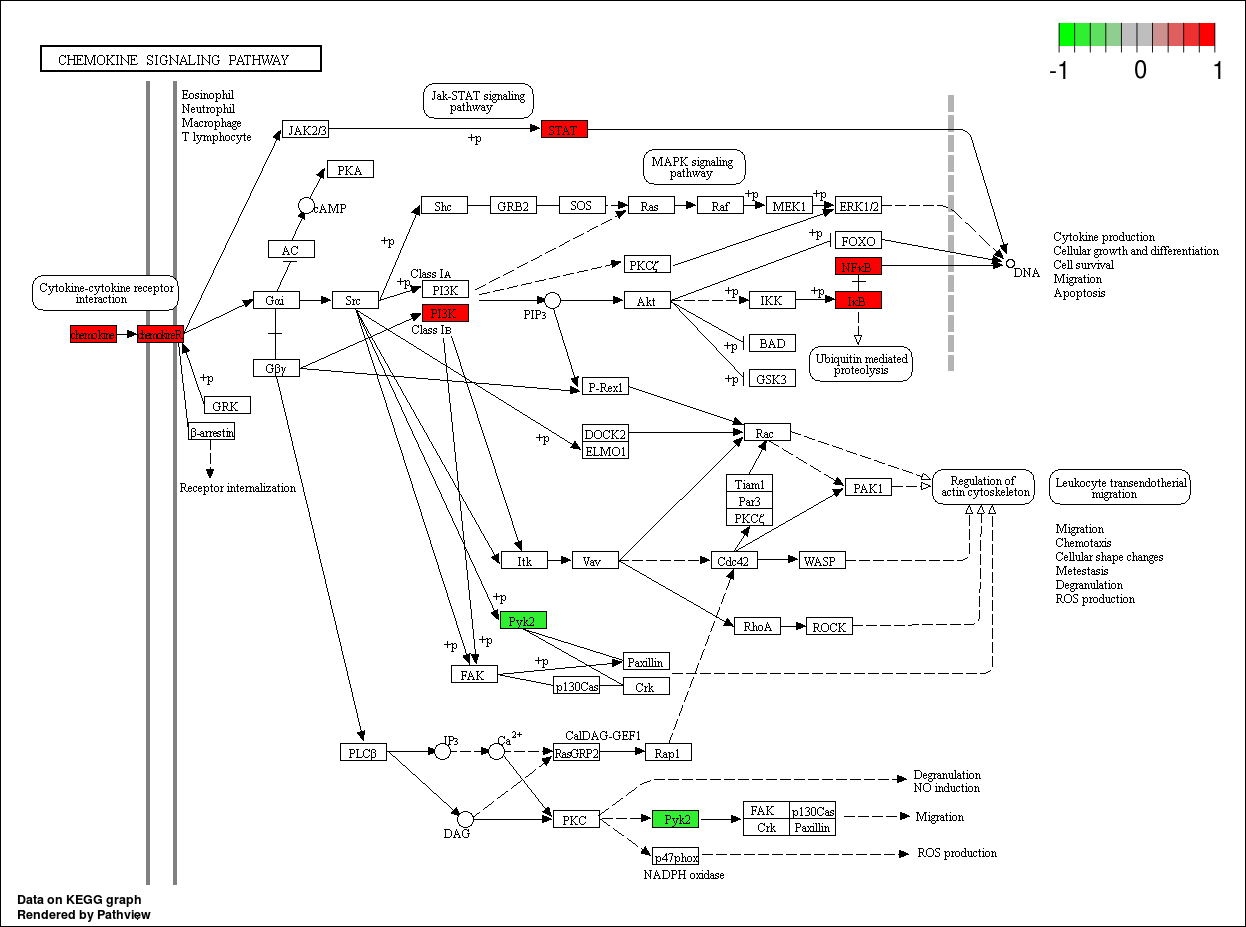

Supplement: Supplementary file 1 [file genes-11-00651-s001.zip › supplementary_materials/Figure_S4.png]

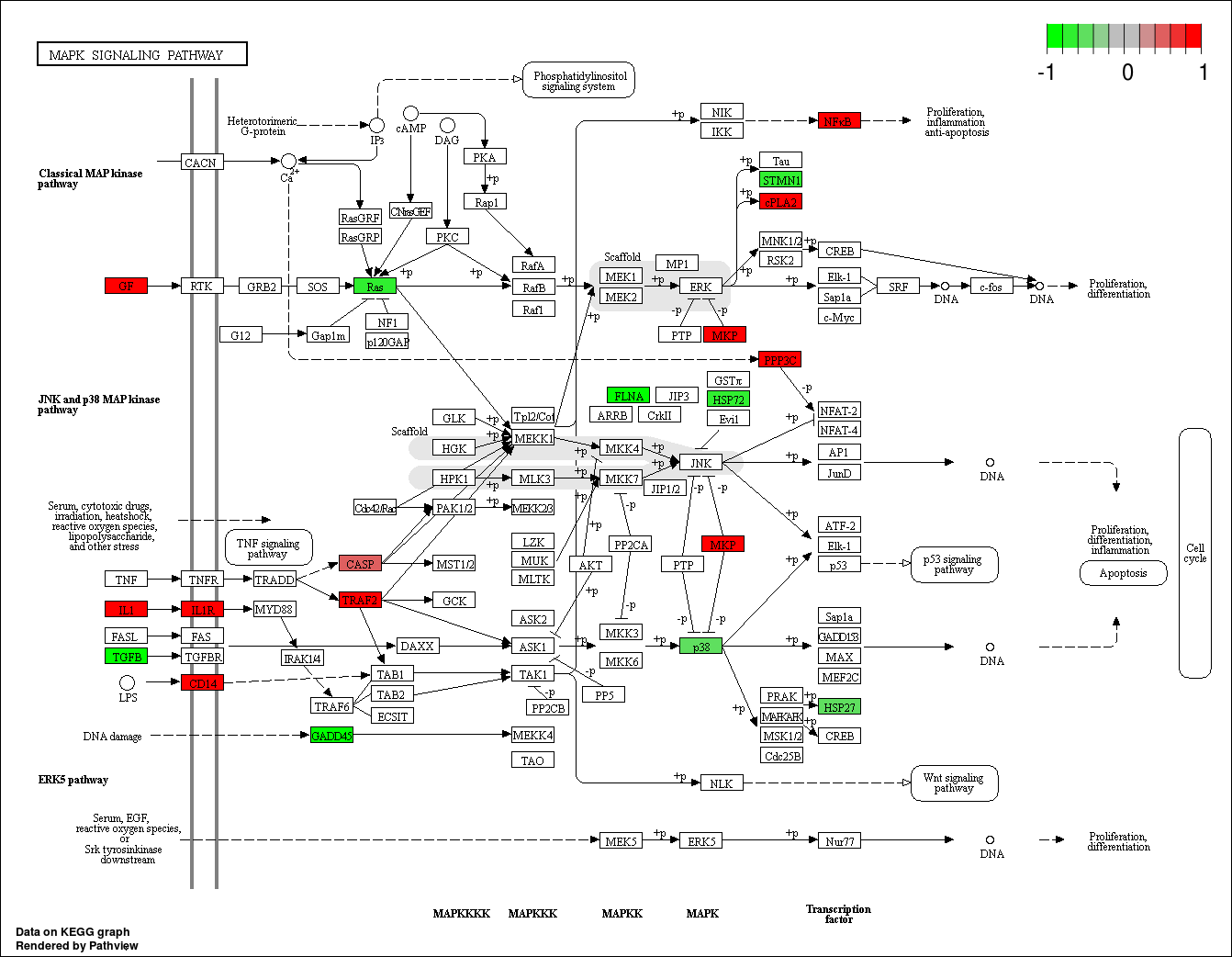

Supplement: Supplementary file 1 [file genes-11-00651-s001.zip › supplementary_materials/Figure_S5.png]

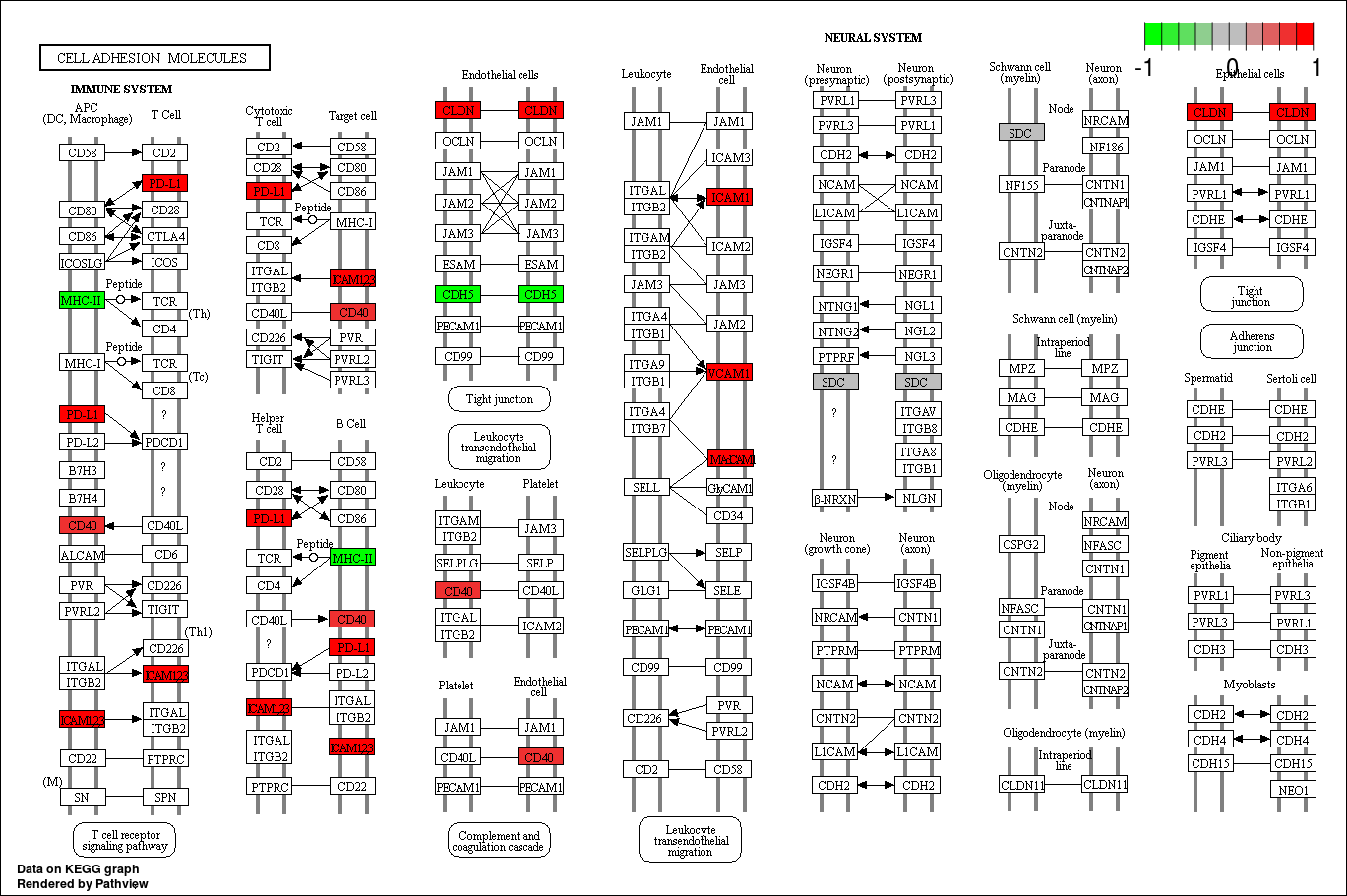

Supplement: Supplementary file 1 [file genes-11-00651-s001.zip › supplementary_materials/Figure_S6.png]

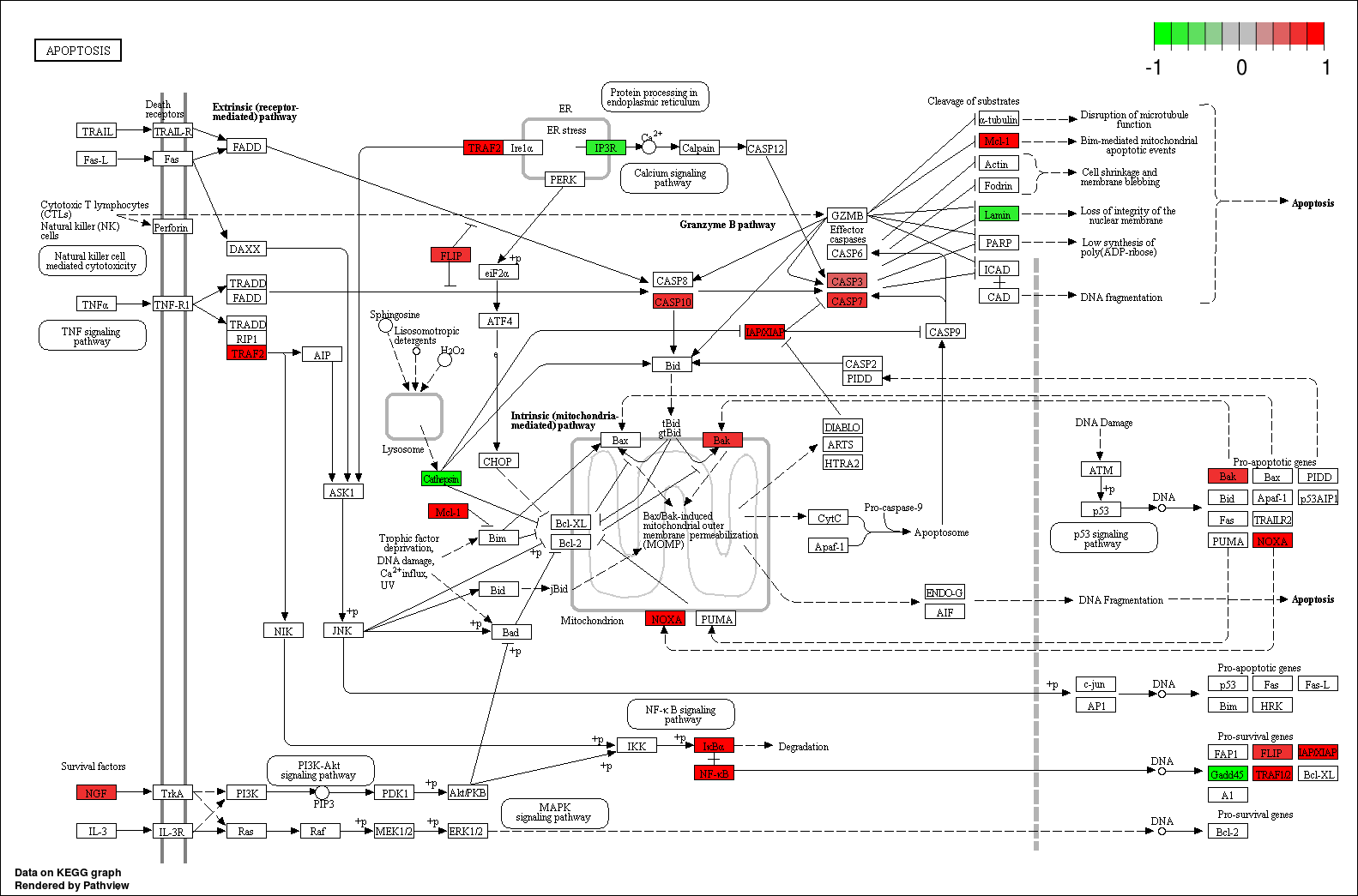

Supplement: Supplementary file 1 [file genes-11-00651-s001.zip › supplementary_materials/Figure_S7.png]

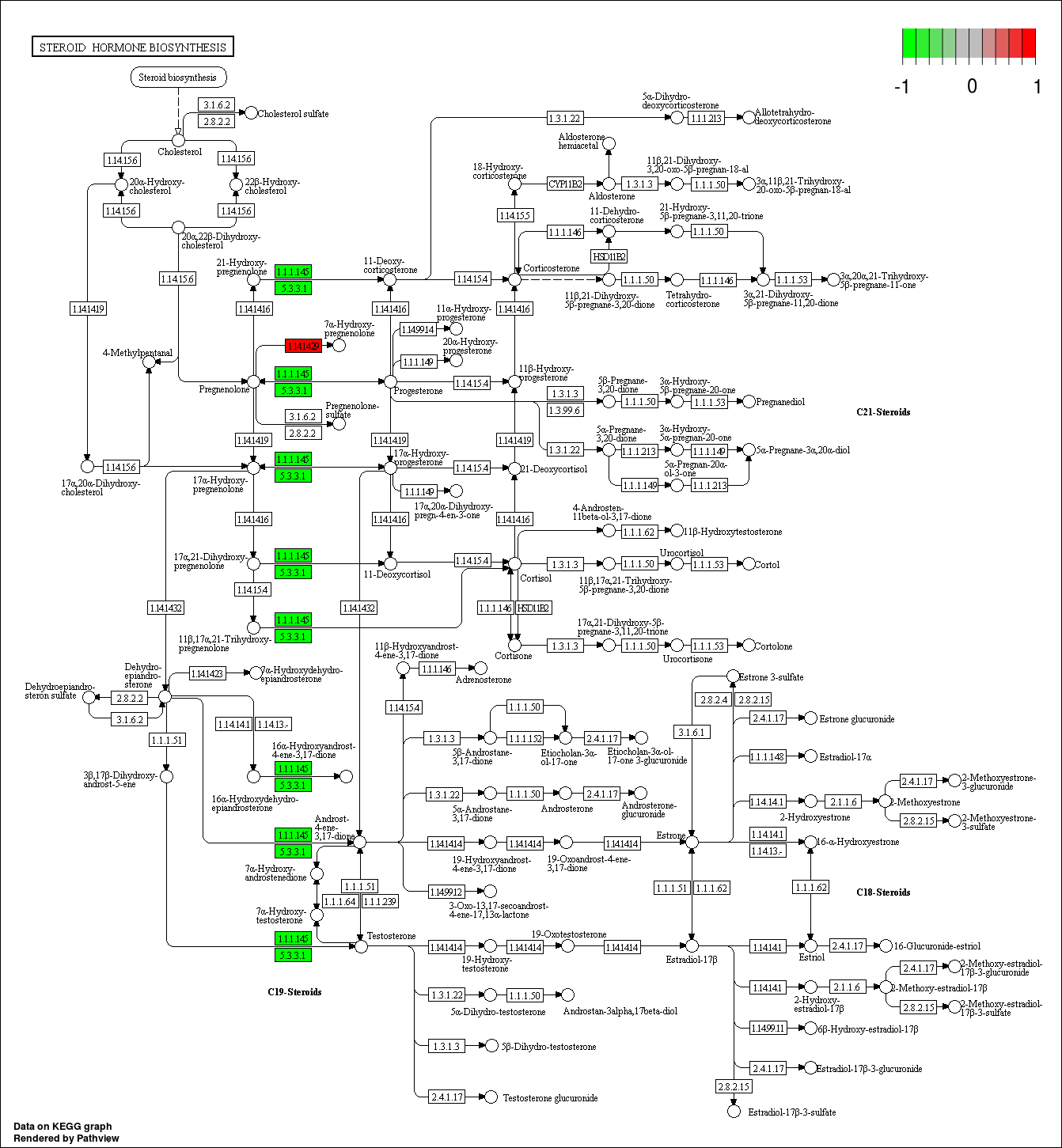

Supplement: Supplementary file 1 [file genes-11-00651-s001.zip › supplementary_materials/Figure_S8.png]

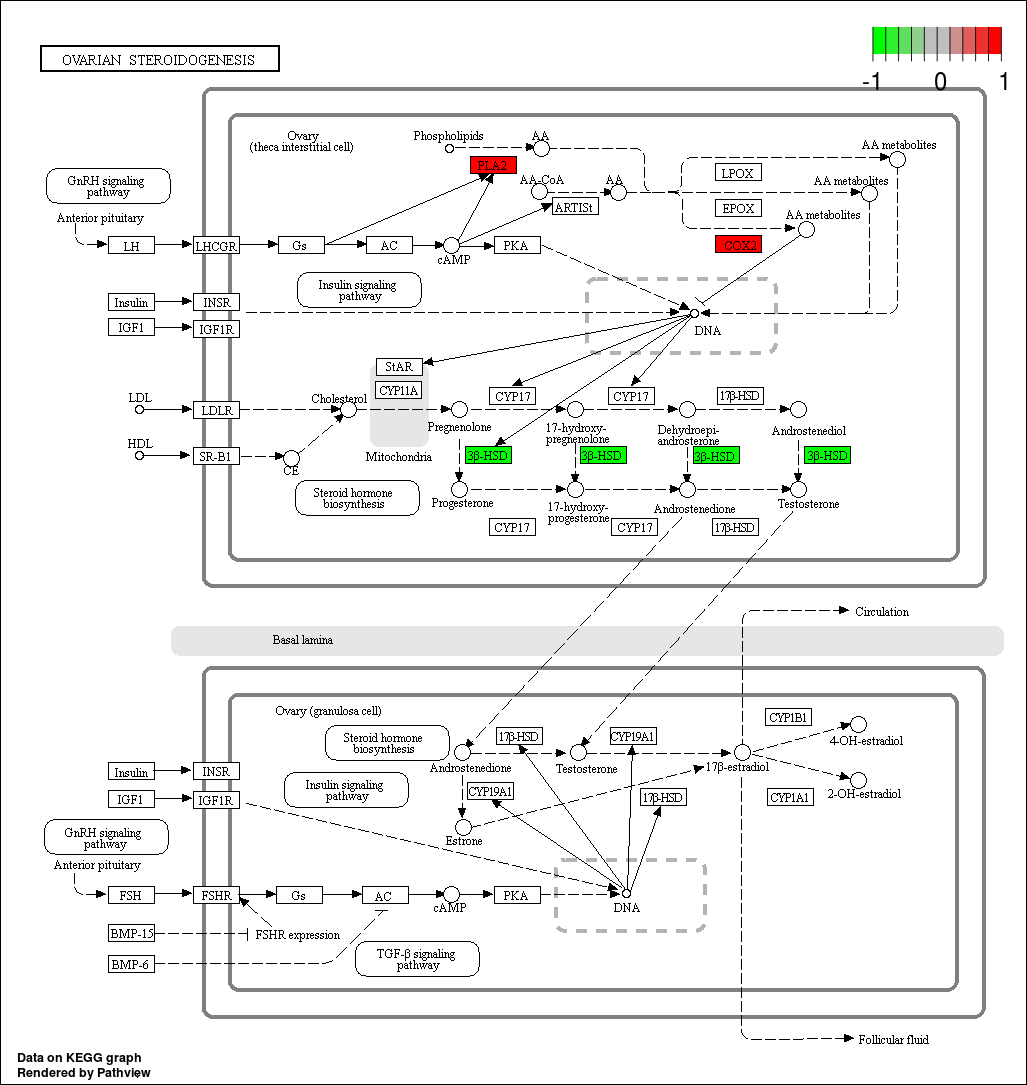

Supplement: Supplementary file 1 [file genes-11-00651-s001.zip › supplementary_materials/Figure_S9.png]
